# Synovial Complement Factors in Patients with Periprosthetic Joint Infection after Undergoing Revision Arthroplasty of the Hip or Knee Joint

**DOI:** 10.3390/diagnostics11030434

**Published:** 2021-03-04

**Authors:** Frank Sebastian Fröschen, Sophia Schell, Matthias Dominik Wimmer, Gunnar Thorben Rembert Hischebeth, Hendrik Kohlhof, Sascha Gravius, Thomas Martin Randau

**Affiliations:** 1 Department of Orthopaedics and Trauma Surgery, University Hospital Bonn, 53127 Bonn, Germany; Matthias.Wimmer@ukbonn.de (M.D.W.); Hendrik.Kohlhof@ukbonn.de (H.K.); Thomas.Randau@ukbonn.de (T.M.R.); 2 Department of Orthopaedics and Trauma Surgery, Ev. Waldkrankenhaus Bonn, 53177 Bonn, Germany; sophia.schell@hotmail.de; 3 Institute of Medical Microbiology, Immunology and Parasitology, University Hospital Bonn, 53127 Bonn, Germany; hischebeth@microbiology-bonn.de; 4 Orthopaedic and Trauma Surgery Centre, University Hospital Mannheim, Medical Faculty Mannheim of the University of Heidelberg, 68167 Mannheim, Germany; sascha.gravius@googlemail.com

**Keywords:** synovial fluid, complement system, periprosthetic joint infection, revision arthroplasty

## Abstract

The role and diagnostic value of the synovial complement system in patients with low-grade periprosthetic joint infection (PJI) are unclear. We sought to evaluate, for the first time, the usefulness of synovial complement factors in these patients by measuring the individual synovial fluid levels of complement factors (C1q, C3b/iC3b, C4b, C5, C5a, C9, factor B, factor D, factor H, factor I, properdin, and mannose-binding lectin [MBL]). The patients (*n* = 74) were classified into septic (*n* = 28) and aseptic (*n* = 46). Receiver-operator characteristic curves and a multiple regression model to determine the feasibility of a combination of the tested cytokines to determine the infection status were calculated. The synovial fluid levels of C1q, C3b/C3i, C4b, C5, C5a, MBL, and properdin were significantly elevated in the PJI group. The best sensitivity and specificity was found for C1q. The multiple regression models revealed that the combination of C1q, C3b/C3i, C4b, C5, C5a, and MBL was associated with the best sensitivity (83.3%) and specificity (79.2%) for a cutoff value of 0.62 (likelihood ratio: 4.0; area under the curve: 0.853). Nevertheless, only a combined model showed acceptable results. The expression patterns of the complement factors suggested that PJI activates all three pathways of the complement system.

## 1. Introduction

Measurement of synovial biomarkers is well established as an additional tool in diagnosing periprosthetic joint infection (PJI). In contrast to systemic inflammatory markers, they are less affected by systemic or chronic inflammatory diseases, other infection sites in the body, or relevant comorbidities such as obesity [1,2,3]. However, sample material cannot be acquired as easily as serum, as sterile joint aspiration is necessary. In the current diagnostic workup for painful arthroplasties, measurement of synovial biomarkers can be considered a standard procedure [4,5].

Nevertheless, a single perfect synovial biomarker that is accurate, reliable, and easy to measure has not been identified yet and might never be found. However, correct identification of a PJI is essential for choosing the right treatment in view of the far-reaching consequences for the patient [6].

The evaluation of new biomarkers is a topic of ongoing research and ongoing debate. A recent meta-analysis by Lee et al. to evaluate the diagnostic accuracy of promising synovial biomarkers, revealed that out of thirteen analyzed biomarkers, α-Defensin in addition to IL-6 and C-reactive protein displayed the best results for diagnosing a PJI [2]. Nevertheless, diagnostic accuracy does not implicate diagnostic utility in routine diagnostic as Kleemann-Forst et al. could show [7,8]. The complement system, as part of the innate immune response against common pathogens, might be a possible target, but so far, it has not been investigated in relation to PJI. It is comprised of >30 different proteins in the plasma and on cell surfaces, organized into a hierarchy of proteolytic cascades, which lead to the generation of proinflammatory mediators, opsonization, and targeted lysis of pathogens) [9]. Previous studies demonstrated its decisive role in patients with rheumatoid arthritis or in the differentiation between with osteoarthritis or traumatic knee injury [10,11]. Nevertheless, it might not only be a valuable tool in diagnosing a PJI, but also be useful in the treatment of patients with a PJI. As we currently lack studies evaluating the role of synovial complement factors in patients with a low-grad PJI, it is unknown if pathway inhibitors, respectively an activation of synovial complement factors might be a possible treatment option.

This study addresses the question of whether synovial complement factors are useful for diagnosing PJI and offer new insights into the inflammatory pathway and activation of the innate immune response in patients with low-grade PJIs.

In this retrospective study, we aimed to evaluate the usefulness of synovial complement factors, using a multiplex protein microarray system in the analysis of joint aspirates from patients who underwent revision surgery for painful total joint arthroplasty (TJA). We hypothesized that measurement of a combination of multiple synovial biomarkers is superior in predicting PJI than measurement of individual markers.

## 2. Materials and Methods

### 2.1. Synovial Fluid Samples

Between January 2015 and December 2017, 74 patients were included in our study. The inclusion criteria were pain persisting for >6 months after TJA of the hip or knee joint and a subsequent need for revision surgery. Formal patient consent was acquired for the use of any material not needed in routine diagnostics for the purposes of this study. The joint aspirates analyzed in this study were all obtained as part of the preoperative and intraoperative diagnostic routine, in accordance with our treatment algorithm [12]. Approval for the study was obtained from the local institutional review board (University of Bonn Ethics Committee, No. 226/13). After puncture of the relevant joint, the acquired joint aspirates were aliquoted and stored until batch analysis, as described elsewhere [5].

The complement factors C1q, C3b/iC3b, C4b, C5, C5a, C9, B, D, H, I, properdin, and MBL were quantified using the Merck MILLIPLEX Human Complement Panel 1 and 2 (Cat. No. HCMP1MAG-19K; Darmstadt, Germany) as described by the manu-facturer. The sample preparation is described elsewhere [5].

All the patients were treated in accordance with our in-house algorithms, depending on the symptom duration and whether PJI was suspected [5,12]. The evaluation of the measured synovial complement factors did not influence the decision-making process regarding the patient’s treatment plan. On the basis of all the available clinical, microbiological, and histopathological data, the patients were assigned to either the “periprosthetic infection” (PJI) or “aseptic revision” (non-PJI) group for the analysis, in accordance with the following modified criteria developed by the Musculoskeletal Infection Society (MSIS) [13]:

A PJI was considered proven when

(1) at least one of the following major criteria was fulfilled:
  - The presence of a sinus tract with evidence of communication with the joint or visualization of the prosthesis
  - Two positive cultures of the same organism
(2) a score ≥6 with the following minor criteria was achieved (preoperative diagnosis):
  - Elevated serum C-reactive protein (>1 mg/dL) or D-dimer level (>860 ng/mL; score: 2)
  - Elevated serum erythrocyte sedimentation rate (>30 mm/h; score: 1)
  - Elevated synovial white blood cell count (>3000 cells/µL) or leukocyte esterase (++; score: 3)
  - Positive synovial alpha-defensin (signal-to-cutoff ratio > 1; score: 3)
  - Elevated synovial polymorphonuclear percentage (>80%; score: 2)
  - Elevated synovial C-reactive protein level (>6.9 mg/L; score: 1)

For patients with inconclusive minor criteria (scores of 2–5; 0–1: not infected) or dry tap, the following operative criteria were used to fulfill the definition of PJI:

(3) A score ≥6 with the following criteria is achieved (intraoperative diagnosis; ≤3, not infected; 4 or 5, inconclusive):
  - Preoperative score
  - Positive histology (score: 3)
  - Positive purulence (score: 3)
  - Single positive culture (score: 2)

### 2.2. Statistical Analyses

Data were collected retrospectively as anonymized data sets from the electronic and paper records in a Microsoft Excel spreadsheet (version 2101, Microsoft Corporation, Richmond, CA, USA). Statistical analysis was performed using SPSS Statistics 25 for Windows (version 25, SPSS Inc, an IBM company, Chicago, IL, USA) and GraphPad Prism 8.0 (version 8.0, GraphPad Software, La Jolla, CA, USA). The statistical significance between the groups was assessed using the Mann–Whitney test. Any probability value of <0.05 was considered statistically significant (alpha value = 0.05). Relative risk and odds ratio, and sensitivity and specificity were assessed with the Fisher exact test. Receiver-operator characteristic curves were constructed to assess the discriminatory strength of the parameters in distinguishing between PJI and non-PJI on the basis of the area under the curve and to determine the sensitivity and specificity of the different cutoff values. The Youden index was calculated to determine the highest sensitivity and specify and the best likelihood ratio. Sensitivity was given preference over specificity when the Youden index was similar over a range of possible cutoff values.

To combine multiple analytes into a single model, all the parameters were assessed for collinearity and predictive power before being combined in a multiple regression model. From among the models with equal power, the simplest one was chosen.

## 3. Results

By using the modified MSIS criteria, all the patients could be assigned to one of the two groups. All the results were conclusive. All 74 synovial fluid samples could be successfully analyzed with the used system.

**Figure 1:**
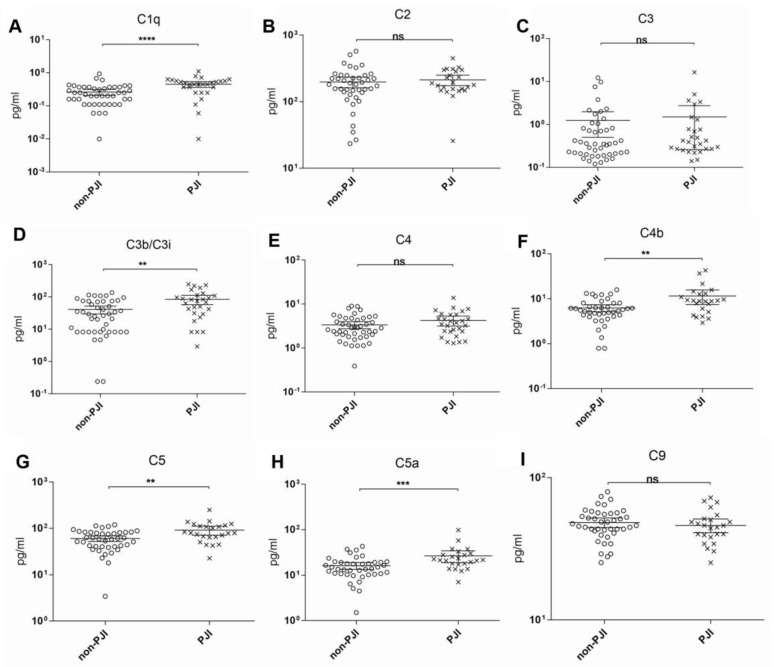
Scatterplots of the measured biomarkers I (**A**–**I**; Statistical significance: ** *p* < 0.01; *** *p* < 0.001; **** *p* < 0.0001; ns: No statistical significance).

**Figure 2:**
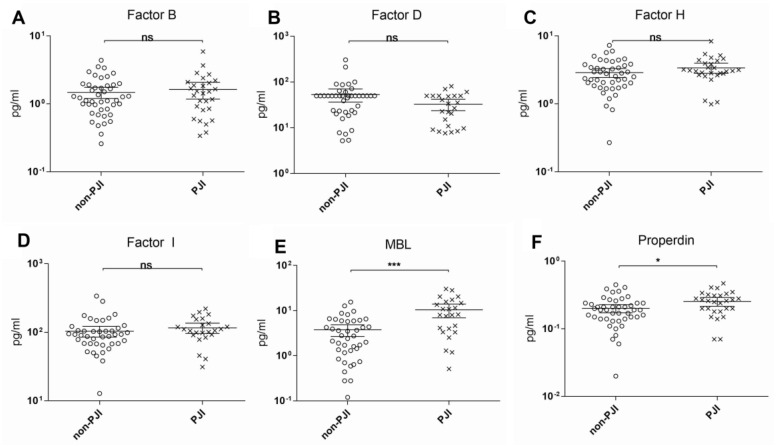
Scatterplots of the measured biomarkers II (**A**–**F**; Statistical significance: * *p* < 0.05, *** *p* < 0.001; ns: No statistical significance).

Figure 1 and Figure 2 depict the scatterplots of all the measured synovial biomarkers. The clinical parameters of all the patients are summarized in Table 1. In 28 cases, PJI could be diagnosed (37.8%), whereas 46 cases did not meet the diagnostic criteria for PJI (62.2%).

Only for C1q, C3b/C3i, C4b, C5, C5a, MBL, and properdin, a significant difference in mean synovial fluid levels was found between the PJI and non-PJI groups. Of all complement factors tested, C1q showed the best sensitivity and specificity, followed by C4b, C5a, and C3b/iC3b. Unfortunately, none of these showed an acceptable performance as an individual biomarker (Table 2).

In addition, properdin and C1q showed a high collinearity. We excluded properdin owing to its inferior sensitivity or specificity in the multivariate linear regression analysis.

**Figure 3:**
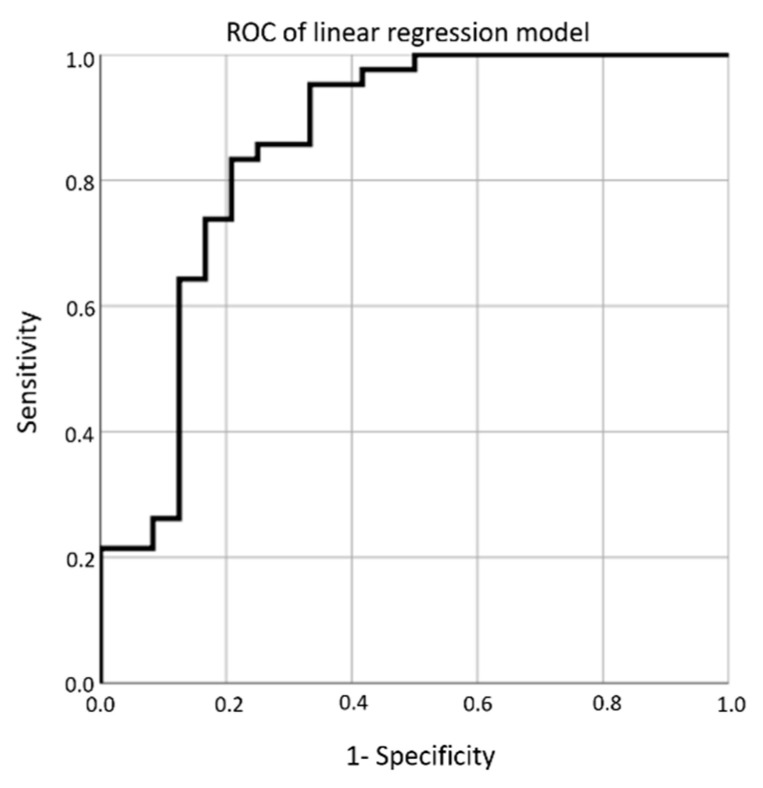
Receiver-operator characteristic (ROC) curve of the multivariate linear regression analysis of C1q, C3b/C3i, C4b, C5, C5a, and MBL.

Multivariate linear regression models were subsequently calculated using the significant complement factors. Regression analysis of the combined complement factors revealed a sensitivity of 83.3% and a specificity of 79.2% for a cutoff value of 0.62, with a likelihood ratio of 4 (AUC: 0.853, 95% CI: 0.745–0.961; *p* = 0.000002; Figure 3; Table 3).

## 4. Discussion

To date, the use of biomarkers has been considered a useful tool for diagnosing PJI [14]. Nevertheless, the biomarkers that should be evaluated remains controversial. The distinction between PJI and an aseptic cause for revision remains a diagnostic challenge with far-reaching consequences for the patient. To our knowledge, no current study has evaluated the usefulness of synovial complement factors both individually and in combination for diagnosing PJI in patients who underwent revision hip or knee arthroplasties.

In contrast to the adaptive immune system (AIS), which is organized around two classes of specialized lymphocytes and is essential for a long-lived immunological memory, the innate arm of our immunity is composed of immunological effectors that provide an immediate, nonetheless nonspecific, immune response. It plays a vital role in host protection. No strict separation is made to the adaptive immune response. The humoral arm of the AIS is closely linked to the complement system, which is needed for adaptive immune response and long-lived immunological memory [9]. Previous studies outlined the effects of the complement system on the selection and maintenance of B1 cells and therefore on the generation of a relevant antibody response at several different levels of B-cell biology [15,16]. In addition to its decisive role in the humoral AIS, several studies outlined its vital role in the T-cell immune response to viral antigens and alloantigens in a mouse model, although comprehensive knowledge is still lacking on the exact pathways. Therefore, the complement system obviously plays an important role in both the innate and adaptive immune responses [9,17,18].

Our most important finding is that measurement of an individual synovial complement factor is not a useful diagnostic tool for differentiation between (low-grade) PJI and non-PJI. This is expected, as the complement system can be activated through different pathways, including the classic, lectin, and alternative pathways [10]. Apart from the limited diagnostic value of the individual complement factors, the results regarding the pathogenesis of PJI and the poorly understood immune response presented herein might be of interest. Most studies focus on cellular and humoral immune responses to pathogens in septic arthritis and PJI [4,14]. This study shows that the innate immune system and complement factors seem to play a role in the local immune response of the joint as well. Few studies have investigated up to this extent, and some preliminary results have hinted at a role of C3a in septic arthritis, but no insights are available on possible interactions with other factors, let alone the more complex constellation of foreign-body-associated joint infections [17,18].

**Figure 4:**
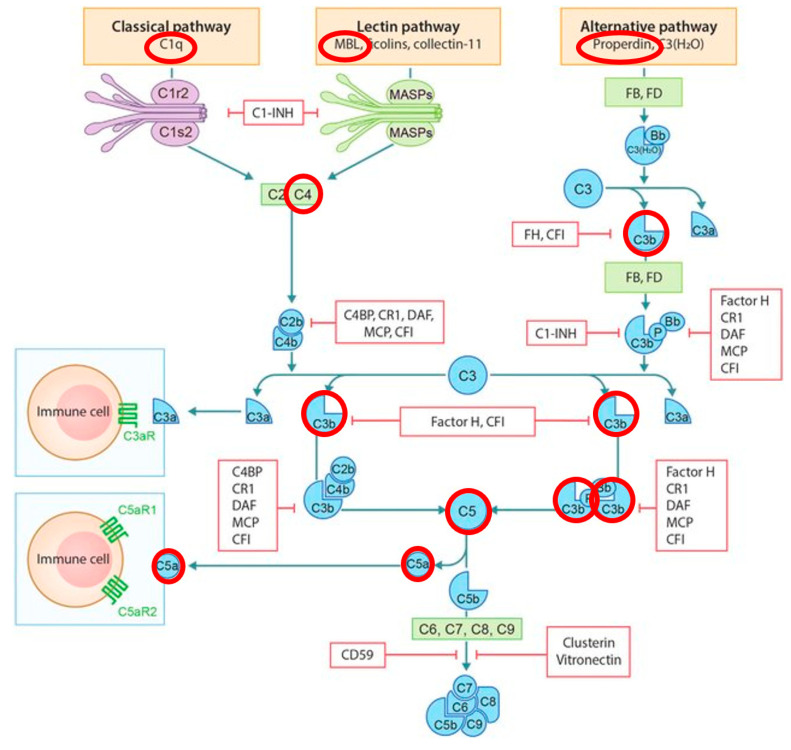
Schematic overview of the complement activation and regulation. Highlighted in red circles are molecules found to be differentially regulated in our study, spread across all three pathways but predominantly in the common final pathway. Adopted from [21]

Figure 4 shows the activation pathways of the complement system, overlaid with the results of our study. We did not find the activation pathway to be the predominantly activated pathway in the patients with low-grade PJI. C1, MBL, and properdin, all differentially regulated between the two groups, play important roles, as they are all pattern recognition molecules of one of the three pathways. Our results, therefore, suggest that in patients with PJI, all three pathways may be activated. Inhibition of the activation cascade was previously discussed as a therapy option in patients with, for example, spondyloarthropathies [19]. Whether such approaches might be beneficial in the treatment of PJI can be further investigated. In addition, our results of elevated synovial fluid levels of C3b/iC3b, C4b, C5, and C5a, which are all part of the final common pathway forming in the end of the membrane attack complex. For diagnostic purposes, the common pathway will therefore be the most sensible aim if measuring all complement factors is not possible. This might be of relevance as Al-IShaq et al. detected elevated C5a levels in a whole blood ex vivo model exposed to biofilm-forming isolates of *Staphylococcus epidermidis* [22].

The exact mechanism of our innate immune response in the case of low-grade PJI remains vastly unknown. The present study only provides a small piece of evidence for the possible role of the complement system and suggests further immunological studies to investigate the possible implications and therapeutic and diagnostic options.

C4A and C9 have been identified in the synovial fluid of patients with ankylosing spondylitis, a rheumatic autoimmune disease, so complement activation is not inevitably tied to pathogen-associated infection but can also be a sign of aseptic inflammation, limiting its diagnostic value in this matter even further [22]. In addition, the prosthesis itself as a foreign body might already have a significant effect on the concentration synovial complement factors. Thordardottir et al. demonstrated a significant activation of the complement system after primary cemented hip arthroplasty [23]. No “normal” complement factor concentration in synovial fluid has been defined. We found measurable amounts of complement factors in almost all samples, which suggest a certain level of activation in any arthroplasty. Comparison of absolute concentrations between studies is difficult because of the wide differences in the assays used. Whether the type or location of the arthroplasty has an effect on the synovial complement factor concentration in patients with or without PJI remains unclear, as no data are available regarding this topic and our sample size was too small to perform an additional subgroup analysis.

Although there are at the moment no studies evaluating the use of synovial complement factors, several studies have evaluated the use of other synovial biomarkers in patients with a PJI with more than acceptable results. Sigmund, respectively Renz et al. described the use of α-defensin in diagnosing a PJI with promising results [24,25]. While Grzelecki et al. evaluated the utility of synovial Calprotectin, with a sensitivity of 93.3% and specificity of 87.5% [26], Quin et al. analyzed the value of synovial and serum IL-6 with more than acceptable results (sensitivity: 94.6%/97.3%; specificity: 92.9%/76.8%) [27]. In comparison to these results several synovial biomarkers will outperform synovial complement factors in diagnosing a PJI. Against this background and in view of our results, synovial complement factors should not replace other biomarkers in diagnosing a PJI, but they still might be a useful tool. Nevertheless, we surely must admit that we are not able to draw a final conclusion regarding the topic of synovial complement factors in patients with PJI as we did not perform a subgroup analysis or an analysis of the cellular immune response to avoid any kind of speculation.

We investigated the aforementioned biomarkers because we believe that detection of (low-grade) PJI is most likely to be successful by reliably measuring the levels of the synovial complement factors as markers of local inflammatory response. However, evaluation of any new diagnostic tool remains complicated because a widely accepted gold-standard definition of the criteria for the diagnosis of PJI is lacking. Owing to their high acceptance, the definition of PJI proposed by Parvizi et al. in 2018 was applied in our study [13].

Our study have limitations, including the small sample size, retrospective analysis, and classification used [13] to assign patients to the PJI and non-PJI groups, which is currently not accepted worldwide as a gold standard. Nevertheless, all the patients could be classified. In addition, the panel was not comprehensive, even across the possible range of complement factors. Therefore, we might have missed a biomarker with a high diagnostic potential.

A combined model of synovial complement factors could show promising results in differentiating between patients with and without PJI. It could therefore be an additional tool for diagnosing PJI. Additional studies with larger samples might be helpful for further evaluation.

## Figures and Tables

**Table 1:** Demographics of the patients in the study collective.

	PJI	Non-PJI	*p* Value ^1^
Total (*n*)	28	46	
F: M	17:11	29:17	0.841
Hip: knee	11:17	10:36	0.104
Age (years)	72.9 ± 11.7	67.6 ± 10.5	0.018
BMI (kg/m^2^)	33.5 ± 11.7	29.7 ± 5.75	0.183

^1^ The p values were calculated using the Mann–Whitney *U* test for body mass index (BMI) and age, and the Fisher exact test for sex. We found patients with PJI to be significantly older than the controls without PJI; otherwise, no significant differences were found between the groups.

**Table 2:** Summary of the results of the receiver-operator characteristic (ROC) analysis of the target cytokines.

Target	ROC Area ^1^	ROC 95% CI	ROC *p* Value	Cutoff (pg/mL)	Sensitivity (%)	Specificity (%)	Likelihood Ratio
C1q	0.754	0.629–0.878	0.00026	0.32	75.0	70.0	2.5
C2	0.560	0.418–0.702	0.424	147.57	79.5	31.0	1.15
C3	0.567	0.434–0.699	0.372	0.27	67.9	47.8	0.97
C3b/iC3b	0.703	0.578–0.826	0.004	41.58	71.4	65.0	2.04
C4	0.587	0.454–0.721	0.210	2.42	0.71	0.46	1.31
C4b	0.689	0.551-0.827	0.011	7.23	70.8	71.4	2.41
C5	0.697	0.566–0.829	0.008	65.09	79.2	59.5	1.85
C5a	0.742	0.619–0.864	0.001	18.05	75.0	69.0	2.41
C9	0.437	0.292–0.583	0.401	39.91	70.8	21.4	0.90
Factor B	0.519	0.381–0.658	0.07	1.09	69.9	50.0	1.39
Factor D	0.305	0.177–0.432	0.009	8.61	83.3	11.9	0.95
Factor H	0.609	0.478–0.741	0.067	2.62	82.1	50.0	1.64
Factor I	0.630	0.488–0.772	0.080	84.84	79.2	52.4	1.65
MBL	0.750	0.623–0.877	0.001	3.80	75.0	59.5	1.84
Properdin	0.652	0.522–0.782	0.029	0.20	75.0	54.3	1.63

^1^ For most targets, no specific cutoff has been reported in the literature. We used the ROC to determine the optimal value with the highest sensitivity and specificity and best likelihood ratio, preferring sensitivity over specificity (CI: confidence interval).

**Table 3:** Summary of the results of the receiver-operator characteristic analysis of the linear regression model.

Cutoff	Sensitivity	Specificity	Likelihood Ratio
0.000	1.000	0.000	1.000
0.023	1.000	0.083	1.091
0.041	1.000	0.167	1.200
0.087	1.000	0.250	1.334
0.114	1.000	0.333	1.500
0.178	1.000	0.417	1.713
0.251	1.000	0.500	2.000
0.364	0.976	0.542	2.129
0.420	0.952	0.583	2.286
0.460	0.952	0.667	2.856
0.491	0.905	0.667	2.713
0.508	0.881	0.667	2.643
0.531	0.857	0.667	2.571
0.574	0.857	0.750	3.429
0.592	0.833	0.750	3.332
0.624	0.833	0.792	4.000
0.656	0.810	0.792	3.886
0.678	0.786	0.792	3.771
0.707	0.738	0.792	3.543
0.742	0.714	0.833	4.286
0.760	0.667	0.833	4.000
0.774	0.643	0.833	3.856
0.799	0.619	0.875	4.951
0.818	0.595	0.875	4.762
0.846	0.548	0.875	4.381
0.851	0.524	0.875	4.190
0.866	0.476	0.875	3.809
0.878	0.429	0.875	3.429
0.890	0.405	0.875	3.237
0.896	0.357	0.875	2.856
0.905	0.310	0.875	2.475
0.908	0.286	0.875	2.286

## Data Availability

The data presented in this study are available in the manuscript.

## Abbreviations

AIS	adaptive immune system
AUC	area under the curve
CI	confidence interval
MSIS	Musculoskeletal Infection Society
PJI	periprosthetic joint infection
ROC	Receiver-operator characteristic
